# Selection of Neurosurgical Applicants in the High-Income Developing Country Lacking Local Residency Program: A Cross-Sectional Study

**DOI:** 10.1055/s-0042-1758832

**Published:** 2023-02-06

**Authors:** Jehad Al-Habsi, Fatema Alhabsi, Sara Al-Jahwari, Tariq Al-Saadi

**Affiliations:** 1 College of Medicine and Health Science, Sultan Qaboos University, Muscat, Oman; 2 Department of Neurology and Neurosurgery, Montreal Neurological Institute, Faculty of Medicine, McGill University, Montreal, Quebec, Canada; 3 Department of Neurosurgery, Khoula Hospital, Muscat, Sultanate of Oman

**Keywords:** neurosurgery selection, Oman, neurosurgery residency program

## Abstract

**Background** Neurosurgery residency became one of the most competitive specialties in the medical field worldwide, which is increasing with time in contrast to the limited positions. Therefore, the requirements for the program have increased. There are different criteria for each program, which are determined by specific factors. It has become increasingly important for medical students to be aware of the factors that affect their acceptance into the program. There was a lack of data regarding the factors that contribute to the selection of neurosurgery residents in Oman

**Methods** A questionnaire composed of 14 questions was conducted, using the SurveyMonkey Web site, among neurosurgeons in Oman which was distributed to the five hospitals that have neurosurgery departments in Oman. SPSS software was used in the analysis of the collected data.

**Results** Forty-four participants responded to the survey. Ninety-five percent of them answered all the questions. Out of all participants, only two were female participants. Standardized international exam scores, such as the United State Medical Licensing Examination and Medical Council of Canada Qualifying Examination, ranked as the most important factor with a percentage of 44, followed by interview performance with a percentage of 33. While the least important factor was the age of applicants, which 46% of the participants ranked 8.

**Conclusion** Most of the participants agreed that standardized exams are the most important factor in the selection of neurosurgery residents followed by interview performance, although there was no significant statistical difference between the two.

Given the increasing numbers of neurosurgery residency applicants and the limited numbers of training positions since 1985, neurosurgery has become one of the most competitive specialties in the United Stated of America. 1 Similarly, in the United Kingdom neurosurgery has ranked in the top 5 most competitive programs for surgical training positions from 2015 up to 2017. 2

When it comes to Oman, there is no local neurosurgical residency program. Applicants interested in neurosurgery need to go abroad for their residency. 3 4 The selection process occurs in two stages; a local selection process followed by a neurosurgery residency match at abroad residency programs. Locally, applicants are selected by a local committee involving neurosurgery consultants. Applicants are requested to provide their curriculum vitae (CV), at least three recommendation letters, pass the Oman Medical Specialities Board (OMSB) exam, and at least one of the following exams: Medical Council of Canada Evaluating Examination, Medical Council of Canada Qualifying Examination 1 (MCCQE1), United State Medical Licensing Examination (USMLE) Step 1 with Step 2, and Australian Medical Council exam with International English Language Testing System scoring 7.0 in average at all sectors or Occupational English Test with an average of B.

Every residency program has different selection criteria for candidate acceptance to their program. These criteria and factors contribute the most to the recruitment of neurosurgery residents which have become the best predictors of residents' performance during their residency years. 1

Burford et al, John et al 16 , and Al Khalili et al all agreed on the importance of medical graduates pursuing neurosurgery residency to have a clear insight of the factors looked at during trainee selection. It is becoming increasingly crucial for medical students to consider these factors, improve their skills regarding them, and build a neurosurgery-focused portfolio starting early during medical school and thus build on their chances to match in the desired program. 1 2 5 This is converting into a double win situation for residency programs and medical graduates, where both are satisfied by their election and thus enhancing productivity in the neurosurgery field.

Medical students interested in neurological surgery need a clear guideline about objective factors influencing match success. Potential applicants in neurosurgery residents rely on available information sources being as program directors, chairmen, faculty mentors, residents, and Web sites to uprise their chances of getting accepted. Nonetheless, this information is based on individualized experiences. Finding objective data can be difficult and initial attempts to gather these data are more difficult because they can easily covey misleading information. 5

To determine significant variables in the selection of neurosurgery residents in Oman this cross-sectional study was conducted. It is aiming to provide advisors and medical graduates with some guidance regarding neurosurgery residents' matches in Oman.

## Methods

A 14-questions survey was developed to identify factors influencing neurosurgery residents' selection. Section 1 collected brief demographic information of the participants. Section 2, participants were asked to order the elements of the current residency application from most important to be ranked (1) to least important to be ranked (7). Section 3, to assess the impact scale of nominated factors on neurosurgery residents' selection by 5-point Likert (1, “strongly agree”; 2, “agree”; 3, “neither agree nor disagree”; 4, “disagree”;5, “strongly disagree”). Furthermore, two questions from section 3 were hybrid linked to two other questions. Participants are transferred only if their answers were strongly agreed or agreed. The questions are as follows: How do you evaluate the applicant's age impact on neurosurgery residents' selection? If the answer was strongly agreed or agreed, the participant is asked to choose whether younger age favors acceptance or older age favors acceptance. The second question was “How do you evaluate the applicant's gender impact on neurosurgery residents' selection?” Participants were asked to specify the gender that favors acceptance if their answer was strongly agreed or agreed.

The survey was created by using the SurveyMonkey Web site. It was distributed to neurosurgeons in Oman and aimed to involve participants from Omani neurosurgery residents abroad and all government hospitals having neurosurgery departments in Oman. These were Khoula Hospital, Sultan Qaboos University Hospital, Sultan Qaboos Hospital in Salalah, Nizwa Hospital, and Sohar Hospital.

The survey was distributed to neurosurgeons through emails, individual social media accounts, social media groups, and text messages to neurosurgeons' phone numbers. In addition, the heads of departments were asked to distribute the survey among the department affiliates. Distribution started in August 2020 and the survey was left open for 5 weeks. At least two reminders were sent over this time interval to gather as many responses as possible.

Statistical analysis was performed using Statistical Package for the Social Sciences (SPSS) software version 23. Descriptive data were summarized using frequency and percentage. Clustered error bars were used to present the ranking of the elements in section 2 of the survey with demographics. A stacked relative bar chart of multiple variables was used to represent the results of the ranking question. On the ground of the small sample size, for the 5-point Likert scale section 1, “strongly agree” and 2, “agree” were combined, 3, “neither agree nor disagree” was considered as a missing value, and 4, “disagree” and 5, “strongly disagree” were combined. Furthermore, residents and medical officers were combined. Combinations were done in pursuance of testing associations between demographic variables and statements in section 3. Associations between variables were tested by chi-square and Fisher's exact test for the 2 × 2 chi-squared test.

## Results

The overall response rate was 44, of whom 39 completed at least 95% of the survey. Of those, 37 respondents completed the survey. All respondents were male participants except two were female participants. Thirteen respondents (33.3%) were consultants, 12 (30.8%) were specialists, 9 (23.1%) were medical officers, and 5 (12.8%) were Omani residents studying abroad. Twenty-four (61.5%) of the participants have more than 10 years of experience in neurosurgery. Fifteen (38.5%) were involved in the selection of neurosurgery residents. Descriptive analysis is shown in Table 1 .

**Table 1:** Descriptive analysis of neurosurgeons who participated in the survey

Characteristic	*n*	%
Gender		
Male	37	94.9
Female	2	5.1
Age		
≤ 35 y	8	20.5
> 35 y	31	79.5
Current job		
Consultant	13	33.3
Specialist	12	30.8
Medical officer	9	23.1
Resident	5	12.8
Years of experience in neurosurgery		
< 5 y	6	15.4
5–10 y	9	23.1
> 10 y	24	61.5
Being involved directly in the selection of neurosurgery residents		
Yes	15	38.5
No	24	61.5

**Fig. 1:**
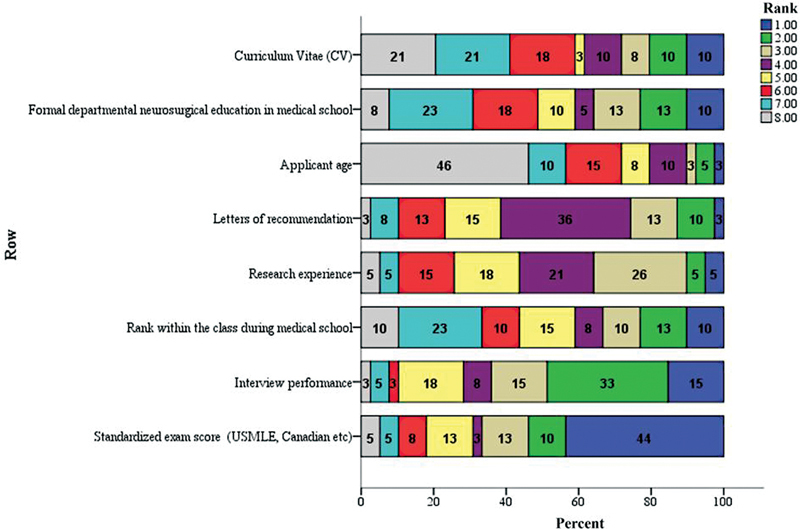
Factors impacting neurosurgery residents' selection ranking.

In section 2, respondents were asked to rank the elements of the residency application from most (1) to least (8) important based on their impact on the selection decision of neurosurgery residents. Results are shown in Fig. 1 . Forty-four percent of the participants ranked standardized international exams such as USMLE, MCCQE, etc. as the number one influencing factor in choosing neurosurgery residents. Two-thirds of the participants who were involved in the neurosurgery residents' selection ranked standardized exam score as one of the most two important factors with a *p* -value of 0.055 as shown in Table 2 . Participants share the same opinion regarding the applicant's rank within the class during medical school and the applicant's age with a *p* -value of 1.000. This was tested using the chi-square test of the most two important factors impacting neurosurgery residents' match selected by participants who were involved in neurosurgery residents.

**Table 2:** The most two important factors impacting neurosurgery residents match based on participants involved in neurosurgery residents' selection

	Chi-square test ( *p* -value)
Standardized exam score	0.055
Curriculum vitae (CV)	0.220
Research experience	0.279
Formal departmental neurosurgical education in medical school	0.437
Letters of recommendation	0.658
Interview performance	0.748
Rank within the class during medical school	1.000
Applicant age	1.000

Ten out of the 13 consultants who are part of this study, representing 76.9%, selected interviews as one of the most two important factors. On the other hand, 49% of the consultants ranked the applicant's age as the least important factor with an average of 6.3.

**Fig. 2:**
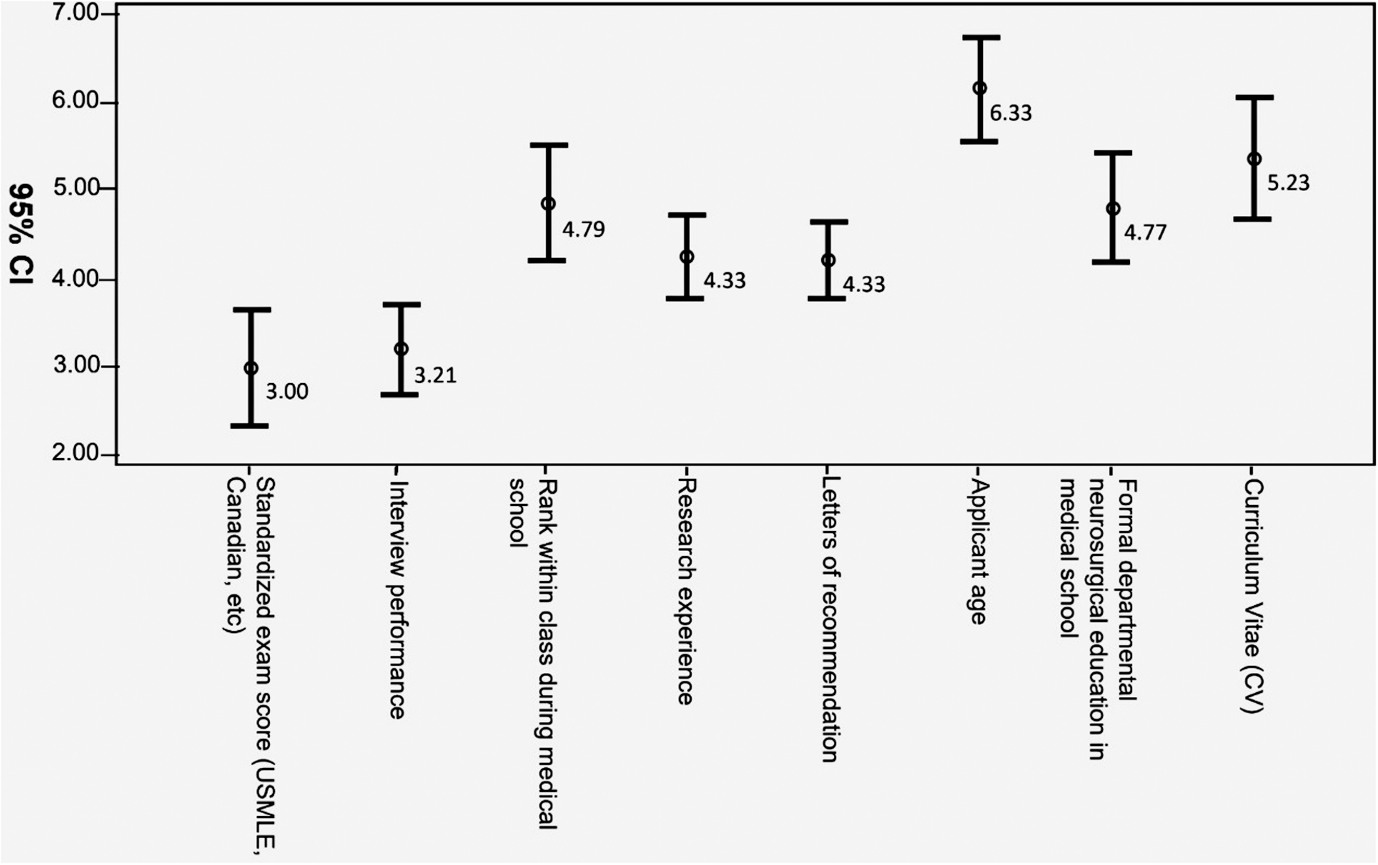
Factors impacting neurosurgery residents matching among all participants.

Standardized exam score was ranked by all the participants at an average of 3 and interview performance was ranked as 3.2 on average. In addition, there was no significant difference between them and the other variables ( Fig. 2 ). It is shown also that there was no difference in the average rank of research experience and letters of recommendation as both were scored as 4.3. On the other hand, the applicant's age was scored on an average of 6.3, which is the least average ranked ( Fig. 2 ).

About 23% of all participants chose the applicant's rank within the class during medical school to be the first or the second most important factor. Similarly, 23.1% of all participants chose formal departmental neurosurgery education in medical school to be an important factor in neurosurgery residents' selection. Only two of the participants who were involved in the selection of neurosurgery residents thought formal departmental neurosurgery education in medical school can be one of the most two important factors playing role in neurosurgery residents' selection. One-quarter of participants with experience exceeding 10 years in neurosurgery rated the applicant's rank within the class during medical school as one of the most two important factors. Half of these participants have been involved in the selection process.

Thirty-six (92%) of all participants thought that applicants' age does not impact their chances to be accepted for the neurosurgery residency program. Only one of those involved directly in the selection of neurosurgery residents disagreed with them. Twenty-five out all participants agreed that younger applicant age favors acceptance, more than half of these participants have experience in neurosurgery exceeding 10 years.

**Fig. 3:**
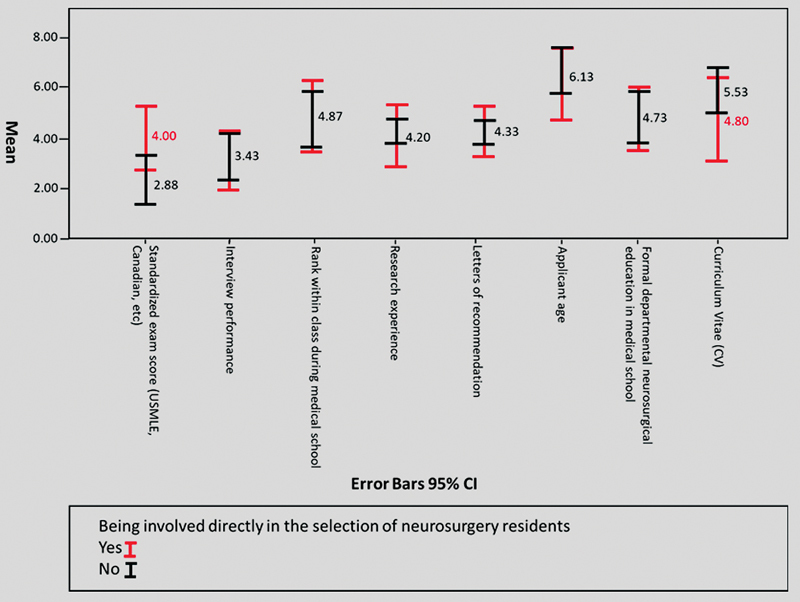
Factors impacting neurosurgery residents matching among participants involved directly in the selection process versus not being directly involved.

Although participants who were not involved in neurosurgery residents' selection value standardized exams score, with an average of 2.8, more than participants who were involved in the selection of neurosurgery residents with an average of 4.0. Fig. 3 shows that there was no significant opinions variation among the two groups.

Twenty of all participants believed that an applicant's gender influences acceptance and 17 of them think that being a male applicant favors acceptance into the neurosurgery residency training program. Moreover, 8 of these had over 10 years of experience in neurosurgery. All of the female participants and two-thirds of male precipitants agreed that the gap years between graduating from medical school and applying for a residency program influences the selection process of candidates. There was no significant difference between participants involved in the selection process and other participants' opinions regarding the impact of the applicant's gender and the gap years on residency match. Almost 94% of all participants agreed that research experience and subsequent publications in bibliometric research impact acceptance. Furthermore, 13 out of the total 14 members involved in residents' selection agreed on the same, but it was statistically insignificant. Moreover, all participants agreed that having anatomical training based on cadaver's dissection in medical school, doing well in ethical/behavioral scenarios, interview performance, number of contiguous ranks work experience, volunteer experiences, neurosurgical knowledge assessment, and the presence of a well-recognized neurosurgeon as a reference alter neurological surgery residency match. Twelve percent only thought that manual dexterity test results do not impact the results of residency match.

## Discussion

Applicants will apply online through OMSB portal. Candidates who are filling the requirements of scholarship as listed previously will be interviewed by the OMSB scholarship committee. Selected candidates are sponsored to do a scholarship in one of these countries: Canada, U.S., Australian, and New Zealand. After that, those candidates are expected to get acceptance from one of the universities with a formed liaison with OMSB. After getting the acceptance, application should be approved by the Ministry of Higher Education, Research, and Innovation in Oman. Currently, there are eight residents and fellows undergoing training in the United States, Canada, France, and Saudi Arabia.

Respondents identified standardized exam scores, interview performance, and letters of recommendation as the most important components of a residency application, which is consistent with previous studies. 1 2 5 6 Based on the results of our survey, 54% ranked standardized exam scores as one of the two most important factors with an average of 3. Forty-four percent rated it as the number one influencer. Two-thirds of participants being involved in the selection of neurosurgery residents ranked standardized exam score as one of the most two important factors with a *p* -value of 0.055.

The standardized exam score is the most important factor in the resident selection process. In comparison to previous studies, Al Khalili et al and Nicholas et al 1 , where it was found that the interview is the most important factor, in our study, it was ranked as the second important factor in the selection process. 1 2 It was observed that standardized exams score in particular (USMLE Step 1) was the second important factor. Although studies have shown the absence of a correlation between (USMLE 1) score and residency performance program directors use it as a filtration mechanism for candidates. 1 2 Gauer and Jackson found that residency specialty match was significantly associated with USMLE Step 1 and USMLE Step 2 Clinical Knowledge (CK) scores, where neurosurgery applicants of graduates of the University of Minnesota Medical School from 2011 to 2015 scored the second-highest score in USMLE Step 2 CK. 7 A literature review of 11 studies regarding the interview performance and its effect on neurosurgery residents' selection showed a positive correlation between residency performance and interview. The positive performance was identified as in-training examinations, licensing board examinations, clinical evaluations, and a composite score or rank of resident performance. Three studies highlighted that interview performance changed the rank of some applicants significantly by moving them either higher or lower (> 10 positions) compared to their preinterview rank. 8

Based on the USA National Residency Match Program data in 2009–2016 analysis it was extracted that in addition to USMLE Step 1 and 2 scores, the number of research projects was a statistically significant independent factor impacting neurosurgery match success. 9 Supported by the conclusion of a literature review of 69 articles highlighting the advocation of creating national and international associations to support medical students and encourage collaborative research involvement. 6 However, the current survey found that the applicant's research experience was ranked as the third factor by 26% of participants and only 4 (10%) chose the research experience of the applicant as one of the most two important factors. Twenty-one (87.5%) participants with experience of more than 10 years in neurosurgery and 12 (57.1%) of them who were involved in neurosurgery residents' selection disagreed with that. This opinion was supported by a study estimating the preresidency publication volume of U.S. neurosurgery interns where neurosurgery interns had at least one publication. It was also found that interns at top-25 neurosurgery residency programs tended to have a higher number of publications than the average number of publications per intern among all programs. 10 The importance of the research experience of applicants was emphasized by a bibliometric analysis conclusion. It was stated that the H-index value is a powerful predictor of matching into neurosurgical research institutions. H-index is a value representing the number of publications, h, cited at least h times. It was observed that H-index correlates strongly with the number of original research articles, along with years since first publication and the impact factor of the journal where the research was published. Moreover, H-index can be improved by early research experience, targeting journals with high impact, and participating in clinical and laboratory investigations. 11 In Oman, research experience is taken into consideration during candidates' selection for scholarships. During the interview applicants are asked about their oral presentation at conferences, poster presentation, and published papers in indexed journals.

These all emphasize the importance of research experience of applicants applying for neurosurgery residency matches.

A survey of 113 program directors in the United States evaluating the value of standardized letters of recommendation emphasized the high weight placed on these letters as they provide a realistic way to compare applicants for interview invitation elections. For that reason, almost two-thirds (65%) of program directors agreed on standardized letters of recommendation implementation would improve the resident selection process and increase the objectivity. 1 In comparison to our survey, it was found that letters of recommendation were ordered in the fourth order by 36% of all participants. While only three (20%) participants involved directly in the selection of neurosurgery residents ranked it as one of the most two important factors. This reflects the diversity of opinions about the value of the impact of letters of recommendation on residents' match. In Oman, application for neurosurgery residency scholarship requires submitting at least three letters of recommendation along with the CV. In this study, a third of participants being involved in neurosurgery residents' selection ranked CV as one of the most two important factors which were not statistically significant. On the other hand, CV evaluation is widely used in residency admission criteria in some countries outside the U.S. overriding local national tests conversely to the U.S. where USMLE board scores stand out as one of the most crucial elements. 6 CV usually highlights extramural activities, community services, and volunteering activities of the applicant which elaborates on the applicants' personality, and their coping mechanisms with life stressors. When studying the independent predictors of long-term satisfaction with the residents' selection, it was discovered that there is a growing importance of extramural activities as an independent predictive of long-term satisfaction in selecting residents. Extramural activities are represented by all extracurricular activities away from medicine. 2

By referring to Stumpo et al's review, it was shown that women were underrepresented in neurosurgery residency programs since 1990. Renfrow et als 17 analysis disclosed that women matching into neurosurgery increased from 10.7% in 1990 to 1999 to 12% in 2000 to 2009. 6 It is worth mentioning that in our study, 71% of participants believed that gender has an influence on acceptance and 17 participants thought that being a male applicant favors acceptance into neurosurgery residency training program.

According to studies done in the U.S., medical school attended was selected as one of the factors affecting neurosurgery residents' selection by neurosurgery residency program directors. 2 Furthermore, Chandra et al 12 highlighted that graduating from small medical schools or schools without an associated neurosurgery department is a disadvantage facing applicants in the U.S. neurosurgery match as it is difficult to stand out among applicants from a brand-name medical school or a strong neurosurgery program. 12

A previous study conducted in Oman revealed that a high number of students believed that adding a neurosurgery rotation during medical school will have a positive impact in the field and contribute to the awareness and knowledge of the neurosurgical conditions. 13 14 This is similar to this survey findings where only one of the participants believed that the absence of formal departmental programs to deliver standardized and effective neurosurgical education should not interfere with neurological surgery match odds. On the other hand, being a “known entity” by a neurosurgery program and neurosurgeons that can provide a strong letter of recommendation may serve as the strongest card for match success mainly for lesser-known applicants. 10 Similarly, our participants believed it is a factor influencing neurosurgery applicants' selection.

Only one of the participants thought that validated online personality assessment tool/personality trait should not alter acceptance possibility in neurosurgery match. Two of the participants thought the same regarding research letters of recommendation. However, six of the participants had a similar opinion regarding letters of recommendation from non-neurosurgical mentors. These findings were similar to the approach that was suggested to mitigate the disruption of the cancellation of neurosurgical subinternships during the coronavirus disease 2019 pandemic and order to minimize its impact on the 2020 to 2021 neurosurgery residency application cycle. It emphasized on letters of recommendation from a neurosurgical faculty, research letters of recommendation, and letters of recommendation from non-neurosurgical mentors commenting on applicants' attributes and interpersonal skills. In addition, it advocated for validated online personality assessment tools as a way to evaluate applicants' personality is and suggested that it may be considered superior to letters of recommendation. 15

Up to date, there is a lack of well-established criteria of neurosurgical residents' selection. However, there are multiple factors influencing the selection of neurosurgical applicants including gender, age, research experience, letters of recommendation, standardized international exams, CV, interview performance, graduating from a medical school with formal departmental neurosurgery program, and rank within the class during medical school.

Provided that this study is limited by the small sample size and the absence of formal criteria for selection of neurosurgery resident as a reference, further studies are recommended to provide medical students and graduates interested in neurosurgery with some guidance regarding neurosurgery residency applications in Oman.

## Conclusion

Our study showed that the factors which affect the acceptance of neurosurgery scholarship candidates in Oman are similar to other countries. Most of the participants agreed that standardized exams are the most important factor in the selection of neurosurgery residents followed by interview performance although there was no significant statistical difference between the two; in contrast to applicants' age, which was selected as the least important factor in the selection of neurosurgery residents in Oman.

**Abbreviations**
USMLE: United State Medical Licensing Examination
MCCQE: Medical Council of Canada Qualifying Examination
SPSS: Statistical Package for Social Science
AMC: Australian Medical Council
IELTS: International English Language Testing System
OET: Occupational English Test
