# Supplementary material for: Selection of Neurosurgical Applicants in the High-Income Developing Country Lacking Local Residency Program: A Cross-Sectional Study
Source: Surg J (N Y). 2023 Feb 6;9(1):e1–7. doi: 10.1055/s-0042-1758832 (PMC9902197; doi:10.1055/s-0042-1758832)
Supplement: Supplementary file 1 — Supplementary Material [file 10-1055-s-0042-1758832-s2100179.pdf]

Supplementary Material Sections 1–3

Section 1

Demographics

1. Gender
   a. Male
   b. Female
2. Age:
   a. Less than 25 years
   b. 25-35 years
   c. More than 35 years
3. What is your current job?
   a. Resident
   b. Medical officer
   c. Specialist
   d. Consultants
   e. Other (please specify)
4. Years of experience in neurosurgery
   a. Less than 5 years
   b. 5-10 years
   c. More than 10 years
5. Have you been involved directly in the selection of neurosurgery residents
   a. Yes
   b. No
6. Current place of work

Section 2

Order the following elements of residency from the most to the least important

- Standardized exam score (USMLE, Canadian, etc.)
- Interview performance
- Rank within the class during medical school
- Research experience
- Letters of recommendation
- Applicant age
- Formal departmental neurosurgical education in medical school
- Curriculum Vitae (CV)

Section 3

5-point Likert scale (1 “strongly agree”, 2 “agree”, 3 “neither agree nor disagree”, 4 “disagree”, 5 “strongly disagree”).
<u>How do you evaluate the following factors impact on neurosurgery residents selection?</u>

Personal information

8. Applicant Gender
9. Which gender favors acceptance
   a. Male
   b. Female
10. Applicant age
11. What is the applicant’s age that favors acceptance to neurosurgery program
    a. Younger age favors acceptance
    b. Older age favors acceptance

 © 2023. The Author(s).

12. Gap years from graduating medical school until applying for the residency program
13. How do you evaluate the following factors impact on neurosurgery residents selection
    - Research experience and the subsequent publications
        - o Bibliometric research (e.g., H-index, i.e., the value that represents the number of publications, H that are cited at least H times)
        - o Total number of neuroscience-related publications
        - o Interview performance
        - o Number of contiguous ranks work experiences, volunteer experiences
        - o Neurosurgical knowledge assessment
        - o Manual dexterity tests (test evaluates ability to grab and place objects quickly)
        - o Ethical/behavioral scenarios
        - o Validated online personality assessment tools/ personality trait

14. How do you evaluate the following factors impact on neurosurgery residents selection
    - Letters of recommendations
        - o Research letters of recommendation
        - o Letters of recommendation from non-neurosurgical mentors who can comment on personal attributes and interpersonal skills
    - Medical school
        - o Availability of formal departmental program to deliver standardized and effective neurosurgical education
        - o Anatomical training based on cadaver's dissection in medical school
        - o Exposure to Neurosurgery during medical school or elective/selective programs
    - References/mentor
        - o The presence of well recognized neurosurgeon as a reference

 © 2023. The Author(s).
